# Enhancing Nurses' Well-Being: Exploring the Relationship between Transformational Leadership, Organizational Justice, and Quality of Nursing Work Life

**DOI:** 10.1155/2023/2337975

**Published:** 2023-11-01

**Authors:** Ibrahim Abdullatif Ibrahim, Ahmed Hashem El-Monshed, Mohamed Gamal El-Sehrawy, Hossam Elamir, Samah Mohamed Abdelrahim

**Affiliations:** ^1^Department of Nursing, College of Applied Medical Sciences, Shaqra University, Shaqra, Saudi Arabia; ^2^Department of Nursing Administration, Faculty of Nursing, Mansoura University, Mansoura, Egypt; ^3^Department of Psychiatric and Mental Health Nursing, Faculty of Nursing, Mansoura University, Mansoura, Egypt; ^4^Department of Nursing, College of Health and Sport Sciences, University of Bahrain, Manama, Bahrain; ^5^Department of Nursing, College of Applied Medical Sciences, Prince Sattam Bin Abdulaziz University, Al-Kharj, Saudi Arabia; ^6^Department of Nursing Administration, Faculty of Nursing, Port Said University, Port Said, Egypt; ^7^Healthcare Management Consultant, ISQua Expert, Ministry of Health, Kuwait City, Kuwait; ^8^Department of Nursing Administration, Faculty of Nursing, Damietta University, Damietta, Egypt

## Abstract

**Aim:**

The study's aim was to examine the relationship between transformational leadership, organizational justice, and nurses' quality of work life.

**Background:**

There is an increasing emphasis on the welfare of nurses and their influence on healthcare services. There is a dearth of comprehensive studies on the influence of transformational leadership and justice within organizations on work-related quality of life. Understanding these connections is critical for improving workplace dynamics and fostering nurse well-being.

**Methods:**

This cross-sectional research included a convenience sample of nurses working in a general hospital. The data were obtained through self-report questionnaires encompassing three reliable and validated scales: the global transformational leadership scale, organizational justice scale, and quality of nursing work-life scale. The data were then analyzed with SPSS 23 and statistical methods (descriptive statistics, Pearson correlation coefficient, and hierarchical linear regression). This study adhered to the STROBE guidelines.

**Results:**

A sum of 527 out of 566 nurses responded to the questionnaires (response rate = 93.1%). The nurses had a high perception of transformational leadership behaviors but had a more moderate view of organizational fairness and the quality of their nursing work life. Transformational leadership was significantly associated with organizational fairness. Also, a positive relationship was found between transformational leadership, justice within organizations, and nurses' quality of work life.

**Conclusions:**

Nurses who consider their leaders as transformative and have the sense of a fair organizational climate are more likely to have higher levels of quality of work life. *Implications for Nursing Management*. Nurse managers should strive to develop transformational leadership behaviors and promote organizational justice to enhance the well-being and satisfaction of nurses in their work environments.

## 1. Introduction

The World Health Organization (WHO) estimates that by 2030, the world will need nine million nurses and midwives to attain the goal of health and well-being [1]. Within the healthcare industry, nurses hold a significant position as they are the frontline caregivers who have a profound impact on patient outcomes and overall healthcare quality [2]. In this context, healthcare organizations require leaders who possess not only a position of authority but also the ability to inspire and guide their teams effectively. The achievement of improved organizational practices in the field of nursing relies on the combined efforts of leadership and staff collaboration. Transformational leadership plays a crucial role in this process, acting as a mediator and yielding favorable outcomes for all parties involved [3].

Transformational leadership is characterized by its capacity to inspire followers to embrace the organization's vision, goals, and strategies actively. This leadership style, marked by its inspirational and motivating nature, has a significant impact on the nursing turnover rate [4]. The implementation of transformational leadership concepts has the potential to impact various aspects of both the workforce and the company. These effects encompass the reduction of burnout, the enhancement of job satisfaction, the retention of nursing staff, and the improvement of overall productivity [5]. As leaders, nurse managers are also essential in fostering a secure, empowering, and gratifying atmosphere for staff members and patients [4].

Transformational leaders tend to promote a fair and equitable work environment, fostering trust, respect, and the provision of adequate resources [6–9]. Nurses working under transformational leaders perceive organizational justice, including distributive justice, procedural justice, and interactional justice, which positively affect their job satisfaction, commitment, and overall well-being [7, 10, 11].

Nurse managers bear the responsibility of maintaining patient safety, promoting quality of care, enhancing quality of work life, and managing the dynamics of change in health care [7]. The leadership/management style is a significant factor influencing nursing job satisfaction and has a direct impact on nurses' quality of work life [12].

Quality of work life is a complex concept that encompasses a range of factors, including possibilities for professional growth, the effective application of talents and abilities, pay, the balance between work and personal life, and the overall state of well-being within the work setting [5].

Transformational leadership is essential to improve productivity at work and quality of life simultaneously. Worker happiness and a sense of justice inside the workplace are also connected to the quality of work life. Nurses' quality of work life and job happiness may improve if managers are fair in their dealings, use inspiring leadership techniques, and make fair decisions [13]. The consideration of leadership's effect on nurses' productivity and working-life quality is of significant importance. Developing a transformational nursing leadership style is a viable organizational approach to enhance nurse performance and foster improved patient care outcomes [12]. The transformational approach of leadership is widely acknowledged as a successful leadership style that may significantly influence several aspects of an employee's professional life, such as career satisfaction, engagement, and performance [2, 11, 14, 15].

Additionally, organizational justice, a critical aspect of workplace dynamics, has a documented association with nurses' health and well-being, as well as positive work-related outcomes [16]. Organizational justice pertains to the perceptions held by employees on the just and equitable treatment they receive within the organization. This concept incorporates various aspects such as equity, fairness, and social ties that are present in the workplace [17]. Nurses who possess a perception of fairness and justice within their respective healthcare organizations are inclined to exhibit behaviors that surpass their prescribed job responsibilities [16, 17].

Organizational justice has been found to have a significant impact on the quality of work life [18–21]. High organizational justice improves work-related outcomes, health, and well-being among registered nurses environment [16, 22–24]. On the other hand, low organizational justice is linked to undesired work-related outcomes and health problems [16, 23, 24]. Nurses who perceive low organizational justice may experience dissatisfaction with their profession and organization, leading to a lower quality of work life.

Furthermore, previous studies have demonstrated a significant association between organizational justice in healthcare environments and the level of job satisfaction among nurses [20, 25]. Berthelsen et al., [26] have also demonstrated a positive correlation between the perception of organizational justice at the unit level and the reported quality of care by staff members. In light of the intricate nature of the present-day healthcare landscape, it is imperative for nurse leaders to accord utmost importance to the concept of organizational justice [16].

Nevertheless, the current body of literature mostly examines transformational leadership, organizational justice, and quality of work life as distinct concepts, failing to acknowledge the potential combined impact they may have on improving the well-being of nurses and the quality of healthcare. The study aims to provide significant findings that can help healthcare leaders, administrators, and policymakers implement strategies to enhance nurse well-being, justice practices within organizations, and overall quality of nursing life at the workplace.

### 1.1. Aim of the Study

The aim of this study is to investigate the relationship between transformational leadership, organizational justice, and the quality of work life in the nursing profession.

### 1.2. The Theoretical Framework

**Figure 1:**
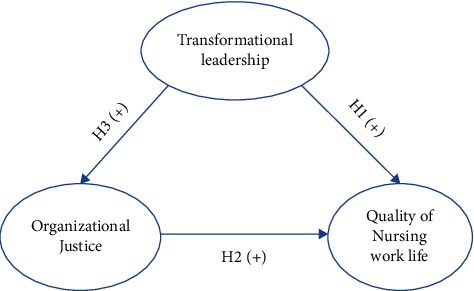
The proposed conceptual model of the study.

The conceptual model employed in this study was founded upon a theoretical framework that has been constructed from the job demands-resources (JD-R) model (Figure 1). The JD-R model provides a framework for examining the impact of job characteristics on employees' well-being [27, 28]. Job demands referred to the physical cognitive or emotional requirement of a job that can be perceived as stressful or challenging for employees. These demands can include high workload time pressure emotional labor and other factors that require effort and may drain employee energy. On the other hand, your job resources are the aspect of the work environment that can help employees to achieve their work goals [27, 28]. According to the job demands-resources model (JD-R), transformational leadership and organizational justice can be viewed as job resources that can enhance the quality of nursing work life by providing support, autonomy, and fairness.

### 1.3. Literature Review and Hypotheses Development

#### 1.3.1. Transformational Leadership and Quality of Nursing Work Life

Transformational leadership is a leadership style characterized by the leader's ability to inspire and motivate their followers to achieve their higher level of performance and personal growth [29]. This leadership approach has been widely studied and recognized as a key determinant of employee satisfaction, commitment, and overall organizational success [10, 30]. In the context of nursing, the transformational leadership style has been founded to positively influence various aspects of nurses' work life and include job satisfaction, organizational commitment, and professional development [2, 3, 7, 12, 21, 31]. Therefore, the following hypothesis developed:

  H1. Higher levels of transformational leadership will be positively associated with improved quality of nursing work life.

#### 1.3.2. Organizational Justice and Quality of Nursing Work Life

Numerous studies examined the relationship between organizational justice and quality of work life. These studies consistently suggested that higher levels of organizational justice are associated with improved work-related outcomes. For instance, Arab and Atan [22] argued that perceived distributive, procedural, and interactional justice all contribute to employee job satisfaction and job performance. Berthelsen et al. [26] demonstrated that the shared perception of organizational justice climate was significantly associated with perceived quality of care and affective commitment to the organization. In the nursing context, the study of Dong et al. [17] revealed that perceived job characteristics and organizational justice can improve nursing care quality through work engagement. We can conclude that nurses are more likely to feel valued and motivated, leading to increased job satisfaction, commitment, and overall positive work outcomes. Therefore, the following hypothesis developed:

  H2: High perceptions of organizational justice will correlate with enhanced quality of nursing work life

#### 1.3.3. Transformational Leadership and Organizational Justice

Transformational leaders are more likely to be perceived as fair and just by their employees. This is because they are seen as role models who exhibit ethical behaviors, treat employees with respect, and involve them in the decision-making process. The leaders that exhibit transformational leadership conduct in a manner that is regarded by their followers as respectful, fair, and aligned with moral and ethical principles might anticipate a higher level of organizational attachment from their followers as a suitable reaction to the provision of interactional justice [32]. The study of Deschamps et al. [33] demonstrated that transformational leaders have a positive impact on their employees' motivation through different aspects of organizational justice. Also, the mediation study of Gillet et al. [31] revealed that transformational leaders influenced positively on nurses' perception of organizational justice. Therefore, the following hypothesis developed:

  H3. Higher levels of transformational leadership will be positively associated with increased perceptions of organizational justice among nurses

The proposed conceptual model of the research was developed to visually clarify the relationship between the study variables. The circular shapes were employed to represent the key study variables, and arrows indicated the directional relationships, with plus (+) denoting positive associations.

## 2. Methods

### 2.1. Study Design and Setting

The present cross-sectional research was conducted at a general hospital that is associated with the Egyptian Ministry of Health institutions. This hospital serves a catchment area with an estimated population of over 300,000 individuals. The hospital offers a wide range of medical services including many disciplines such as internal medicine, obstetrics and gynecology, surgery, pediatrics, ophthalmology, orthopedics, dermatology, and psychiatry. In addition, the facility provides diagnostic services including laboratory testing, radiography, and ultrasound. The healthcare system in Egypt is pluralistic, with public and private providers offering diverse services. The government ensures universal health coverage, while private services are accessible to those with financial means. The study follows the STROBE reporting guidelines [34].

### 2.2. Participants

The study utilized a convenience sample strategy due to its practicality and ability to recruit the participants efficiently. The study included registered nurses who are experienced nurses with at least one year in their current nursing position, full-time employed, and directly involved in patient care. Nursing interns and nurses who held management positions or served as shift leaders were excluded from this study. The Egyptian nursing syndicate categorizes registered nurses based on their level of education and qualifications. These categories encompass nurses with technical education, comprising nurses holding a diploma or institute nursing degree (referred to as technical nurses), as well as those possessing a bachelor's degree and postgraduate degree (referred to as specialist nurses).

### 2.3. Data Collection Procedure

The study employed self-administered hard-copy questionnaires to collect data. The initial page of the anonymized questionnaire included the aim of the study and clear instructions on how to complete the surveys, and the deadline for receiving questionnaires was set a week to encourage timely responses and emphasize confidentiality. Following distributing a questionnaire to a group of nurses, the researchers reminded the participants about the significance of their responses. They explicitly requested truthful answers and guaranteed the nurses that the gathered data would be exclusively utilized for scientific research endeavors. Additionally, the researchers checked each questionnaire immediately upon receipt from the nurses after a limited time to avoid missing data. The data collection period spanned from February 10, 2023, until April 25, 2023.

### 2.4. Measures

The questionnaires had three scales that assessed the research variables and the demographic features of the nurses.

This study's demographic factors included age, gender, marital status, nursing categories, and experience.

The researchers utilized the global transformational leadership scale created by Carless et al. [35] to assess the perception of transformational leadership exhibited by nurses' direct leaders/managers. The scale comprises seven items designed to assess four dimensions: two items measure idealistic impact, two measure inspirational motivation, two items measure individual consideration, and one item measures intellectual stimulation and critical thinking. The value of Cronbach's alpha found in this research was 0.87.

The organizational justice scale was developed by Niehoff and Moorman [36] to assess nurses' perceptions of fairness within healthcare organizations. The scale consists of twenty items categorized into three primary dimensions of justice: (1) distributive justice, which encompasses five items; (2) interactional justice, consisting of nine items; and (3) procedural justice, which includes six items. The Cronbach's alpha coefficients for the constructs in this study were as follows: organizational justice (Cronbach *α* = 0.94), distributive justice (Cronbach *α* = 0.87), interactional justice (Cronbach *α* = 0.90), and procedural justice (Cronbach *α* = 0.75).

The quality of nursing work-life scale was initially developed by Brooks [37] to evaluate nurses' subjective perception of the quality of life at their workplace. The scale comprised a total of forty-two items, which were classified into four distinct subscales: (1) work life/home life, consisting of seven items; (2) work design, consisting of ten items; (3) work context, consisting of twenty items; and (4) work world, consisting of five items. The study reported Cronbach's alpha coefficients for various constructs as follows: quality of nursing work life (Cronbach *α* = 0.81), work life/home life (Cronbach *α* = 0.80), work design (Cronbach *α* = 0.75), work context (Cronbach *α* = 0.87), and work world (Cronbach *α* = 0.71).

The nurses were asked to indicate their degree of agreement with sixty-nine questionnaire items. They were given a 5-point Likert scale, ranging from one represented “strongly disagree” to five represented “strongly agree.”

The evaluation of the study variables was determined by calculating the average score of each variable, which was obtained by dividing the total score by the number of items within the corresponding scale and subscales. The approach utilized in this study involved the conversion of original ratings into a standardized Likert scale, which encompassed a range from 1 to 5. Hence, scores falling within the range of 1–2.59 signify a low level of perception, while scores ranging from 2.60 to 3.39 suggest a moderate level of perception. On the other hand, scores falling within the range of 3.40–5.0 signify a high level of perception [38].

The researchers adhered to a translation and back-translation methodology for the English version of the scales that aligned with established practices observed in previous studies [39–42]. The researchers translated the study scales from English to Arabic and compared the translated texts to generate an initial version. An English teacher translated the scales back into English, and a native English speaker reviewed it to ensure it matched the original version. A panel of five specialists evaluated the Arabic version of the scales to assess readability, clarity, meaningfulness, and face validity. The final version was validated after incorporating revisions suggested by the reviewers. Translating the scales into Arabic acknowledges participants' linguistic proficiency in their native tongue, reduces language barriers, enhances cultural relevance, respects linguistic diversity, and improves data accuracy. This method aligns with research best practices and emphasizes the need for a careful translation process for validity and reliability.

The scales included in our research, namely the global transformational leadership scale, organizational justice scale, and quality of nursing work-life scale, were subjected to a thorough evaluation of content validity following the principles outlined by Davis [43]. The content validity of all scales was found to be strong, with scores of 1.00, 0.97, and 0.98, respectively. The subscales present in these instruments likewise demonstrated values over the threshold of 0.90, while the individual items had an item-content validity index that topped 0.80. The obtained results provide confirmation of the meticulous construction and validation process of our instruments, confirming their pertinence and suitability for assessing the constructs of our study [43]. Before data collection, the researchers conducted a pilot study that included 25 nurses to assess the research framework and methodologies and address any potential challenges. The pilot study data were excluded from the total study population. Based on the recommendations provided by the pilot study sample and experts' input, the rating scale for assessing the quality of nursing work life was modified from a six-point Likert scale to a five-point Likert scale. This modification was implemented based on the belief that the five-point Likert scale would offer enhanced clarity and facilitate more effective respondent comprehension and response.

### 2.5. Ethical Considerations

The participants were provided with information on the voluntary nature of their involvement, the assurance of anonymity in their replies, and the option to withdraw from the study at any point. Their choice to participate did not affect their professional occupation. Participants provided verbal consent before distributing a questionnaire, and they further solidified their consent by signing an informed consent form. The study received ethical approval from the College of Nursing Ethics Committee at Mansoura University, Egypt, with reference number [P.0397].

### 2.6. Statistical Analysis

The statistical analysis was performed using IBM SPSS Statistics, version 23. The normality assumption was accepted based on the central limit theory as a sample size greater than 30 participants [44]. The descriptive statistics included demographic characteristics and study variables. Categorical variables were provided as frequency and percentage, while continuous data were represented by mean and standard deviation. The study employed Pearson correlation coefficients analysis to investigate the associations among transformational leadership, organizational justice, and quality of nursing work life. The study utilized hierarchical linear regression analysis on two occasions. The initial hierarchical linear regression analysis, referred to as model 1, was used to identify factors associated with quality of nursing work life (dependent variable). The first step included the demographic variables. Transformational leadership was added in the second step (independent variable) and organizational justice in the third step (independent variable). The second hierarchical linear regression (model 2) was conducted to ascertain the factors that are linked to organizational justice (dependent variable). The initial stage involved including demographic data to mitigate their influence. The inclusion of transformational leadership occurred during the second phase (independent variable). The categorical variables were recoded as dummy variables (control variables).

Before conducting hierarchical linear regression, the research variables underwent mean-centering before multiplication. This was carried out to mitigate the issue of multicollinearity throughout the analysis and uphold the assumption of independence of errors among the variables. The possible presence of multicollinearity for the regression model was assessed through tolerance (model 1: 0.17–0.98; model 2: 0.17–0.96) and the variance inflation factor (model 1: 1.03–6.02; model 2: 1.04–6.03), confirming that the basic requirements of regression analysis were satisfied. Statistical significance was set at an alpha level of 0.05 for inferential data analysis.

## 3. Results

Out of a total of 566 nurses, 527 nurses completed the survey resulting in a response rate of 93.1%. The mean age of the nurses included in the study was 33.23 years, with a standard deviation of 7.18 years. Most of the nurses were female (96.2%), married (85.2%), and had a technical degree of education (67.9%). The mean experience year of the nurses was 11.79, with a standard deviation of 7.95 (Table 1).

The mean score of transformational leadership was found to be 3.42 (SD = 0.70). The mean organizational justice score was 2.93 (SD = 0.65). In terms of the subscales of organizational justice, the mean scores for distributive justice, interactional justice, and procedural justice were 2.97 (SD = 0.73), 2.90 (SD = 0.76), and 2.94 (SD = 0.66), respectively. The mean score of quality of nursing work life was 3.29 (SD = 0.44). The subscales work life/home life, work design, work context, and work world received mean scores of 2.97 (SD = 0.59), 3.39 (SD = 0.45), 3.49 (SD = 0.53), and 2.77 (SD = 0.76), respectively (Table 2).

Positive relationships were observed between transformational leadership and organizational justice and its subscales. There is a positive correlation between transformational leadership and the quality of nursing work life and the various subscales. There was a favorable relationship between organizational justice and the quality of nursing work life and its subscales (Table 3).

Hierarchical regression model 1 explains 24.9% of the variance in organizational justice. The strongest predictor of organizational justice was transformational leadership (*β* = 0.468, *p* < 0.001), followed by age (31–40 years) (*β* = −0.133, *p* = 0.021) and nursing categories (*β* = 0.112, *p* = 0.006). This indicates that technical nurses who perceived their leaders as transformational were likelier to report higher organizational justice levels. Also, nurses aged 31–40 years had a significant negative association with organizational justice, suggesting that they perceived lower levels of justice compared to those aged more than 40 years. Hierarchical regression model 2 explains 51.7% of the variance in the quality of nursing work life. The highest predictor of quality of nursing work life was organizational justice (*β* = 0.451, *p* < 0.001), followed by transformational leadership (*β* = 0.350, *p* < 0.001). This means nurses who had their leader as transformational and experienced elevated levels of organizational justice were more likely to report a higher quality of nursing work life (Table 4).

## 4. Discussion

This study aimed to explore the relationship between transformational leadership, organizational justice, and quality of nurses' work life. Additionally, the study aimed to evaluate the variables under investigation.

The study's findings provided empirical support for hypotheses 1 and 2, demonstrating that transformational leadership and organizational justice emerge as substantial predictors of the quality of nursing work life. This underscores the pivotal role these factors play in shaping nurses' experiences and job satisfaction. These findings may be due to different assumptions; first, transformational leadership fosters a positive and supportive work environment where nurses feel valued and motivated. This enhances the overall quality of nurses' work life by promoting job satisfaction and engagement [2, 12, 23]. Second, organizational justice enhances trust and respect among employees, as they believe that the organization treats them fairly [9]. Trust and respect are vital components of a high-quality work life, leading to stronger work relationships and job satisfaction [22]. Third, fostering a positive work environment and promoting perceptions of justice can contribute to the mitigation of burnout and turnover intentions among nurses. Consequently, this enhances the overall quality of the nursing work environment [24].

The study aligns with Akar and Ustuner's [6] research, which found a positive correlation between teachers' perceptions of transformational leadership by school administrators and their work-life quality. This relationship was mediated by their perceptions of organizational support and justice. The study conducted by Gillet et al. [31] revealed that transformational leadership directly affects the quality of work life for nurses and this effect is mediated by justice. In a study, Kasim and Aldarmaki [45] investigated the connection between justice, quality of work life, and turnover intention among police personnel in the United Arab Emirates. Their findings suggested that the quality of work life acts as a mediator in the relationship between justice and turnover intention.

The study by Totawar and Nambudiri [13] discovered that organizational justice has a positive relationship with quality of work life and that this relationship was mediated by job satisfaction. The study by Prameswari et al. [46] found that organizational justice and transformational leadership positively and significantly impact job satisfaction, which is a crucial aspect of the overall quality of nursing work life. Kamel et al. [47] observed a significant positive correlation between the quality of nursing work life, organizational justice, and citizenship behaviors. Moghimi et al. [19] examined the correlation between organizational justice and quality of work life in public organizations in Qom Province, Iran. The study revealed a positive association between distributive, procedural, and interactional justice, and quality of work life.

The study's findings support the cognitive evaluation theory, which suggests that transformational leadership promotes intrinsic motivation in nurses by meeting their needs for autonomy, competence, and relatedness [48]. Transformational leaders empower nurses by granting them autonomy in decision-making, fostering professional growth opportunities, and creating a supportive work environment. These studies have shown that nurses' intrinsic motivation and job satisfaction are improved, leading to a higher quality of work life [12, 19, 49].

The study's results supported the third hypothesis, revealing a favorable link between transformational leadership and organizational justice, which is consistent with previous research conducted in numerous settings and nations [10, 30, 31, 33, 50–52]. Transformational leaders tend to advocate for fairness and transparency and encourage participative decision-making, which are essential components of organizational justice [8]. When nurses perceive their leaders as transformational, they are more likely to feel that decisions are made fairly and that their voices are heard, leading to a higher level of organizational justice perception [31]. Transformational leadership behaviors of nurse managers, such as inspiring, stimulating, considering, and influencing their staff nurses, have been associated with positive outcomes for nurses, patients, and organizations. The results involve diverse aspects including favorable work settings, employee behaviors, fairness in the workplace, safety culture for patients, and contentment with the job [11, 15, 53, 54].

The study found that nurses demonstrated a high perception of transformational leadership, suggesting the presence of positive and inspiring leadership behaviors in their work environment. Furthermore, the participants' perception of justice was at a moderate level. This suggests that there exists an ascertain level of fairness and equity within the workplace, but there may be areas that need to be improved. Additionally, the study showed that nursing work life exhibited a moderate quality from nurses' perspectives. These findings indicate that nurses' work conditions, job satisfaction, and well-being were moderately satisfactory indicating potential for improvement in their work-related experiences. These findings indicate that nurses' work conditions, job satisfaction, and well-being are moderately satisfactory, indicating potential for improvement in their work-related experiences. These findings align with prior studies conducted by Gillet et al. [31]; Sürücü [52]; Akdemir [50]; and Khan et al. [51], which reported high transformational leadership perceptions between nurses and other participants.

Regarding organizational justice, the study by Sürücü [52] aligns with our findings, indicating moderate levels of organizational justice. In contrast, Gillet et al. [31], Moghimi et al. [19], and Yasir et al. [30] reported divergent findings, indicating that nurses exhibited low levels of justice perception and experienced an unfavorable quality of work life. The literature presents diverse findings regarding the quality of nursing work life. Kamel et al. [47] observed a high level of work-life quality and organizational justice. Conversely, Akar and Ustuner [6] discovered a moderate work-life quality and organizational justice level.

### 4.1. Limitations of the Study

This study had some limitations. First, the utilization of a cross-sectional design in this study presents limitations in establishing causal relationships between variables. Second, the utilization of convenience sampling in this research may potentially add selection bias and restrict the ability to generalize results to a broader population of nurses. Finally, it is crucial to recognize that the study was carried out solely inside the confines of a singular general hospital, which may potentially restrict the generalizability of the findings to alternative healthcare settings.

### 4.2. Further Research

In the light of limitations mentioned previously, there are several avenues for further research that can enhance the depth and the breadth of our understanding in this area. Future research should consider employing longitudinal or experimental designs to better establish causal relationships between the variables of interest. Furthermore, conducting an inquiry into the fundamental mechanisms and potential moderators of these associations can offer a more profound comprehension of how to optimize the nursing work environment and boost the quality of patient care. Also, the forthcoming studies should utilize random sampling procedures, such as simple random sampling or stratified random sampling, and include multiple healthcare settings to overcome selection bias and restriction of generalizability of results to a broader population of nurses in the other healthcare settings.

## 5. Conclusions

The study discovered that nurses had a high perception of transformational leadership and moderate levels of organizational justice and quality of nursing work life. Moreover, a positive correlation existed between transformational leadership and organizational justice, indicating that these factors significantly improve the quality of work life for nurses. Those who view their leaders as transformational and work in a fair organizational climate are more likely to report higher levels of quality of work life.

## 6. Implications of the Study

Hospital administrators should invest in developing transformational leadership training programs for nurse leaders to promote a fair and supportive work environment. Additionally, efforts to improve organizational justice and fairness in resource allocation can lead to enhanced nurse well-being and overall job satisfaction. Therefore, nurse managers can enhance the work environment for nurses and yield benefits for both employees and the organization by prioritizing these factors.

## Figures and Tables

**Table 1:** Demographic characteristics of the studied nurses (*N* = 527).

Variables	*N* (%)
Age (years)	
20–30	212 (40.2)
31–40	233 (44.2)
>40	82 (15.6)
Mean (SD)	33.23 (7.18)
Gender	
Male	20 (3.8)
Female	507 (96.2)
Marital status	
Unmarried	78 (14.8)
Married	449 (85.2)
Nursing categories	
Technical nurse	358 (67.9)
Specialist nurse	169 (32.1)
Experience years	
1–5	150 (28.5)
6–10	116 (22.0)
>10	261 (49.5)
Mean (SD)	11.79 (7.95)

**Table 2:** Descriptive statistics of the study variables (*N* = 527).

The study variables	Min-max	Mean (SD)
Transformational leadership	1.43–5.00	3.42 (0.70)
Organizational justice	1.30–4.45	2.93 (0.65)
Distributive justice	1.00–5.00	2.97 (0.73)
Interactional justice	1.00–4.89	2.90 (0.76)
Procedural justice	1.00–4.33	2.94 (0.66)
Quality of nursing work life	2.12–4.43	3.29 (0.44)
Work life/home life	1.29–5.00	2.97 (0.59)
Work design	2.00–4.70	3.39 (0.45)
Work context	2.00–4.60	3.49 (0.53)
Work world	1.00–5.00	2.77 (0.76)

**Table 3:** Correlation matrix among the study variables (*N* = 527).

	1	2	3	4	5	6	7	8	9	10
(1) Transformational leadership	1									
(2) Organizational justice	0.480^*∗∗∗*^	1								
(3) Distributive justice	0.413^*∗∗∗*^	0.800^*∗∗∗*^	1							
(4) Interactional justice	0.463^*∗∗∗*^	0.952^*∗∗∗*^	0.637^*∗∗∗*^	1						
(5) Procedural justice	0.396^*∗∗∗*^	0.899^*∗∗∗*^	0.601^*∗∗∗*^	0.807^*∗∗∗*^	1					
(6) Quality of nursing work life	0.582^*∗∗∗*^	0.632^*∗∗∗*^	0.561^*∗∗∗*^	0.596^*∗∗∗*^	0.527^*∗∗∗*^	1				
(7) Work life/home life	0.330^*∗∗∗*^	0.539^*∗∗∗*^	0.507^*∗∗∗*^	0.489^*∗∗∗*^	0.456^*∗∗∗*^	0.719^*∗∗∗*^	1			
(8) Work design	0.338^*∗∗∗*^	0.383^*∗∗∗*^	0.307^*∗∗∗*^	0.393^*∗∗∗*^	0.296^*∗∗∗*^	0.748^*∗∗∗*^	0.473^*∗∗∗*^	1		
(9) Work context	0.635^*∗∗∗*^	0.547^*∗∗∗*^	0.470^*∗∗∗*^	0.517^*∗∗∗*^	0.469^*∗∗∗*^	0.915^*∗∗∗*^	0.521^*∗∗∗*^	0.552^*∗∗∗*^	1	
(10) Work world	0.301^*∗∗∗*^	0.510^*∗∗∗*^	0.505^*∗∗∗*^	0.462^*∗∗∗*^	0.407^*∗∗∗*^	0.646^*∗∗∗*^	0.399^*∗∗∗*^	0.397^*∗∗∗*^	0.442^*∗∗∗*^	1

^*∗∗∗*^ *p* < 0.001.

**Table 4:** Predictors of organizational justice and quality of nursing work life.

Steps and predictors	Model 1: Quality of nursing work life				Model 2: Organizational justice			
	Unstandardized coefficients (*β*)	Std. Error	Standardized coefficients (*β*)	*t*	Unstandardized coefficients (*β*)	Std. Error	Standardized coefficients (*β*)	*t*
*Step 1*								
Age (reference: >40 years)								
20–30	−0.0180	0.152	−0.088	−1.188	−0.186	0.189	−0.092	−0.987
31–40	0.060	0.093	0.030	0.642	−0.269	0.116	−0.133	−2.321^*∗*^
Gender (reference: female)								
Male	0.016	0.162	0.003	0.098	0.202	0.201	0.039	1.001
Marital status (reference: married)								
Unmarried	−0.056	0.087	−0.020	−0.648	−0.152	0.108	−0.054	−1.404
Nursing categories (reference: specialist nurse)								
Technical nurse	0.037	0.070	0.017	0.537	0.239	0.086	0.112	2.767^*∗∗*^
Experience (reference: >10 years)								
1–5	−0.042	0.143	−0.019	−0.293	−0.095	0.178	−0.043	−0.532
6–10	−0.192	0.110	−0.080	−1.741	−0.047	0.138	−0.019	−0.341

*Step 2*								
Transformational leadership	0.350	0.035	0.350	10.037^*∗∗∗*^	0.468	0.038	0.468	12.215^*∗∗∗*^

*Step 3*								
Organizational justice	0.451	0.035	0.451	12.788^*∗∗∗*^				
*R*, *R*^2^, Adj. *R*^2^, *F*, *p*	0.725, 0.525, 0.517, 63.472^*∗∗∗*^				0.510, 0.260, 0.249, 22.757^*∗∗∗*^			

^*∗*^ *p* < 0.05, ^*∗∗*^*p* < 0.01, ^*∗∗∗*^*p* < 0.001.

## Data Availability

The data that support the findings of this study are available upon request from the corresponding author.
